# Calcitonin Induces Bone Formation by Increasing Expression of Wnt10b in Osteoclasts in Ovariectomy-Induced Osteoporotic Rats

**DOI:** 10.3389/fendo.2020.00613

**Published:** 2020-09-08

**Authors:** Chen-Yuan Hsiao, Tien-Hua Chen, Tzu-Hui Chu, Yen-Nien Ting, Pei-Jiun Tsai, Jia-Fwu Shyu

**Affiliations:** ^1^National Defense Medical Center, Graduate Institute of Medical Sciences, Taipei, Taiwan; ^2^Department of Surgery, Landseed International Hospital, Taoyuan, Taiwan; ^3^School of Medicine, Institute of Anatomy and Cell Biology, National Yang Ming University, Taipei, Taiwan; ^4^Department of Surgery, Trauma Center, Veterans General Hospital, Taipei, Taiwan; ^5^Division of General Surgery, Department of Surgery, Veterans General Hospital, Taipei, Taiwan; ^6^Department of Biology and Anatomy, National Defense Medical Center, Taipei, Taiwan; ^7^Department of Critical Care Medicine, Veterans General Hospital, Taipei, Taiwan; ^8^Department of Psychiatry, National Defense Medical Center, Tri-Service General Hospital, Taipei, Taiwan

**Keywords:** calcitonin, Wnt10b, osteoporosis, osteoclasts, ovariectomy

## Abstract

Calcitonin is a small peptide hormone secreted from the parafollicular cells of the thyroid gland in response to an increase in serum calcium. The inhibition of osteoclastic resorption is the main mechanism by which calcitonin quickly decreases circulating calcium levels. Although calcitonin pharmacologically acts on osteoclasts to prevent bone resorption, the results of studies on genetically modified animals have shown that the physiological effect of calcitonin is in the inhibition of osteoblastic bone formation. Because the calcitonin receptor is only expressed in osteoclasts, the effect of calcitonin on osteoblasts maybe indirect and mediated via osteoclasts. Wnt ligands are involved in various aspects of skeletal biology, including bone remodeling and endochondral bone formation. Wnt10b has recently been recognized as a clastokine, and is potentially a therapeutic target for treating bone disorders. However, the extent to which Wnt signaling is involved in bone physiology and disease is not yet fully understood. We hypothesize that calcitonin indirectly increases osteoblastic bone formation by inducing Wnt10b expression in osteoclasts. Micro-CT analysis revealed reduced bone loss in calcitonin-treated ovariectomized rats. The serum of animals treated with calcitonin had decreased TRAP5b and CTX-1 but increased osteocalcin, P1NP, and Wnt10b. Immunohistochemistry staining showed that the level of Wnt10b in the femur was increased in calcitonin-treated groups as compared with control groups. Hematopoietic mononuclear cells were separated from rat femur and tibia bone marrow, and were induced into osteoclasts following treatment with M-CSF and RANKL. In these cells, immunoconfocal microscopy and Western blot analysis showed that calcitonin induced an increase in Wnt10b expression. In a culture of osteoblasts isolated from neonatal rat calvariae, the calcitonin-treated osteoclast supernatant showed an increase in mineralization, as indicated by ALP and alizarin red staining. Taken together, these results indicate that calcitonin induces bone formation by increasing the expression of Wnt10b in osteoclasts in ovariectomy-induced osteoporotic rats. The present study provides in-depth information about the effects of calcitonin on bone remodeling and will thus help in the development of future potential therapeutic strategies for postmenopausal osteoporosis.

## Introduction

The secretion of calcitonin, a 32 aa peptide hormone, from the parafollicular cells of the thyroid gland is induced by increased serum calcium (1) leading to rapid reduction in circulating calcium levels, mainly through the inhibition of bone resorption. The binding of calcitonin to its receptors on osteoclasts causes a series of major reactions within minutes, including loss of the ruffled border, cell retraction, and the suppression of cell motility and bone resorption (2). It is evident that the pharmacological function of calcitonin is to inhibit bone resorption through lowered levels of circulating calcium; nevertheless, the physiological role of calcitonin remains unclear. Previous studies have found that bone mineral density and calcium metabolism were not influenced in patients either with excess endogenous calcitonin (e.g., those with medullary thyroid carcinoma) or with undetectable circulating calcitonin (e.g., those who had undergone thyroidectomy) (3, 4). Because the fluctuation in serum calcitonin levels does not have any obvious pathological outcomes, it has been suggested that calcitonin should have no physiological role in mammals. This theory is, however, not widely accepted, and the existing consensus is that calcitonin plays a significant role in protecting the skeleton under circumstances of calcium stress (5, 6).

In addition, research on genetically modified animals has demonstrated that the physiological role of calcitonin in bone cells could be in the inhibition of bone formation, in contrast to its pharmacological function of inhibiting bone resorption. The high bone mass attributed to increased bone formation has been found in both Calca KO and Calcr KO mice (7, 8), even though Calcr did not manifest in osteoblasts. Naot and colleagues suggested that the skeletal phenotype of an osteoclast-specific Calcr KO could enhance bone formation (6), similar to that of the global Calcr KO; thus, such findings have solved the previously mentioned contradiction. In short, calcitonin probably has indirect effects on osteoblasts that are mediated via osteoclasts.

Osteoporosis, characterized by remarkable losses of bone mineral density and strength, results in fragility fractures and subsequent high morbidity and mortality (9). During bone remodeling, the bone resorption exerted by osteoclasts in the bone matrix has the capacity to activate osteoblastic bone formation through a coupling reaction. This coupling process ensures the succession of bone formation to bone resorption in the remodeling cycle. Recent studies of the regulatory mechanisms for the cross-talk between osteoclasts and osteoblasts have identified several bone formation-stimulating osteoclast-derived factors (i.e., clastokines) and matrix-derived growth factors, and the authors have asserted that these factors may contribute to the future design of novel osteoanabolic compounds (10). Uncoupling anti-resorptive (e.g., calcitonin, odanacatib, and saracatinib) would be better drugs because they could inhibit osteoclastic bone-resorbing activity while maintaining the bone formation attributed to sustained communication between osteoclasts and osteoblasts (9). A recent study revealed that calcitonin only inhibited bisphosphonate-induced osteoclast apoptosis, and the combined usage of calcitonin and bisphosphonate increased the efficacy of bisphosphonate on bone formation in a rat model of osteoporosis (11). This result implies that different kinds of anti-resorptive agents may induce distinct clastokines. Wnt proteins are usually involved in various aspects of bone biology, including osteoblastic, and osteoclastic functions as well as endochondral bone formation (12, 13). For example, Wnt10b was recently identified as a clastokine, and a potential novel therapeutic target of postmenopausal osteoporosis. However, the role of Wnt ligands in skeletal physiology and disease is not fully comprehended. Therefore, we hypothesize that calcitonin increases bone formation by inducing Wnt10b expression in osteoclasts.

To acquire appropriate data and verify our hypotheses, a number of valid techniques were employed in this study. In ovariectomized rats, the effects of calcitonin on the protection of bone loss and Wnt10b expression were determined by micro-CT, bone histomorphometry, and immunohistochemistry analysis. In osteoclasts obtained from M-CSF- and RANKL-treated hematopoietic mononuclear cells isolated from rat femur and tibia bone marrow, the expression of Wnt10b was determined by ELISA, Western blot, and confocal microscopy analysis. Bone formation analysis was performed in osteoblasts isolated from neonatal rat calvariae and cultured with the calcitonin-treated osteoclast-conditioned medium.

The present study provides evidence that calcitonin induces bone formation by increasing the expression of Wnt10b in osteoclasts and offers further information about the involvement of calcitonin in bone remodeling at the molecular level. These findings will help in future potential therapeutic studies of postmenopausal osteoporosis.

## Results

### Calcitonin Alleviates Bone Loss in Ovariectomy-Induced Osteoporotic Rats

**Figure 1:**
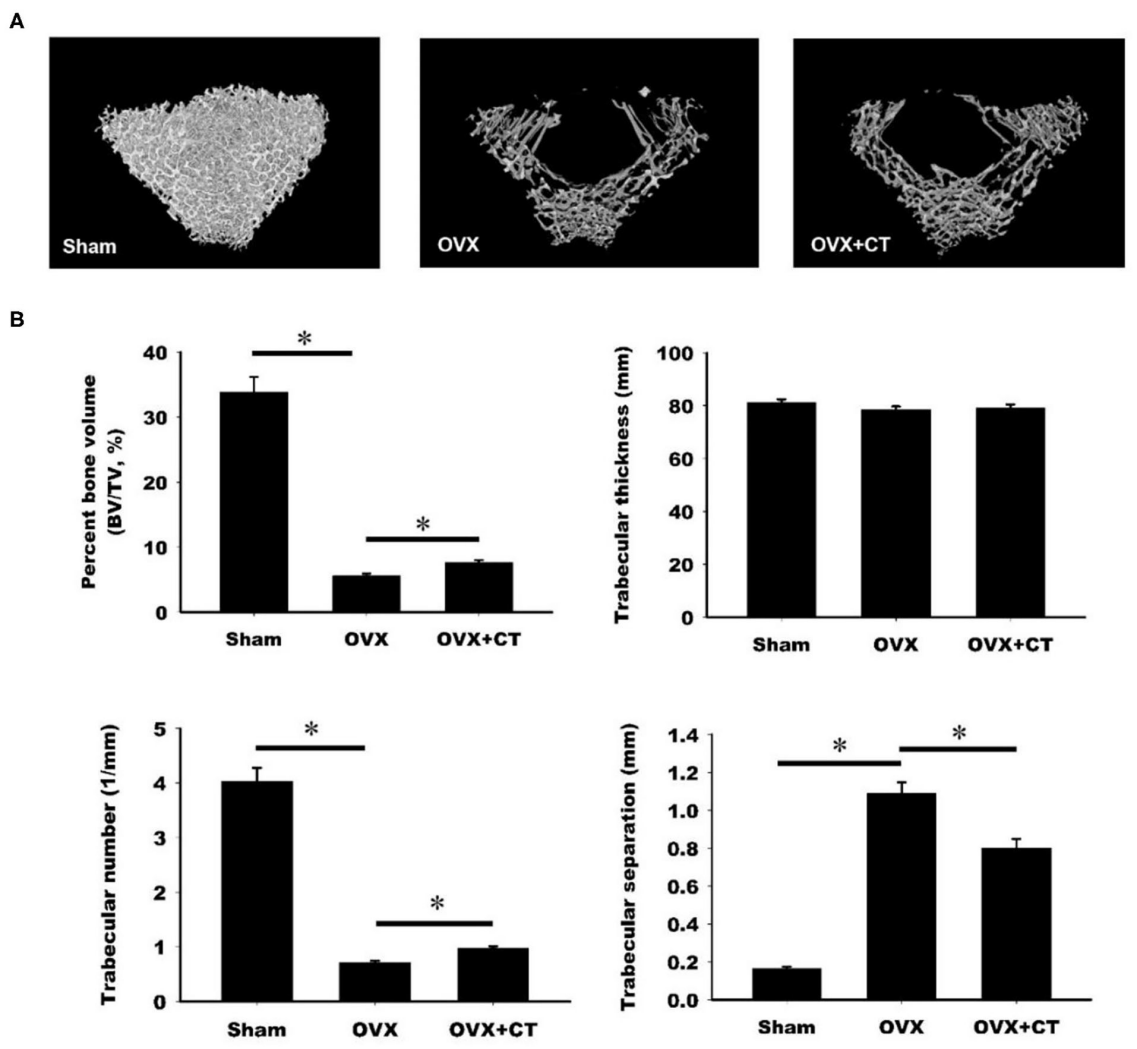
Calcitonin increases femoral trabecular bone in ovariectomized osteoporotic rats. **(A)** Upper panel: micro-computed tomography analysis of the femoral bones in sham-operated rats and ovariectomized (OVX) rats treated with saline or calcitonin (CT, 5 IU/kg/day) five times per week for 4 weeks. Figures are representative reconstructed 3D images from each treatment group. **(B)** Lower panel: quantitative results of the experiment shown in **(A)**. *Indicates a significant difference (*p* < 0.05). *N* = 6 in each group.

The effect of calcitonin on bone deposition was determined in osteoporotic rats 4 weeks after ovariectomy (OVX; Figure 1). OVX or a sham operation was performed in 4-month-old female Sprague–Dawley rats. After 4 weeks, OVX rats received a normal saline or calcitonin treatment for four additional weeks, after which the 6-month-old rats were sacrificed and underwent micro-CT analysis of the femoral bone (Figure 1). Although the 2D images also included cortical bone, the regions of interest containing trabecular bone in metaphysis were selected for subsequent quantification (Figure 1A). Quantitation of these results (Figure 1B) indicated that OVX led to significant bone loss, increased trabecular separation, and decreased trabecular number compared with the control sham operation. Compared with saline, calcitonin treatment significantly increased the percent bone volume and trabecular number in OVX rats. A significant decrease in trabecular separation was found in the calcitonin treatment group.

### Calcitonin Decreases TRAP5b and CTX-1 but Increases Osteocalcin, P1NP, and Wnt10b Serum Levels in Ovariectomy-Induced Osteoporotic Rats

**Figure 2:**
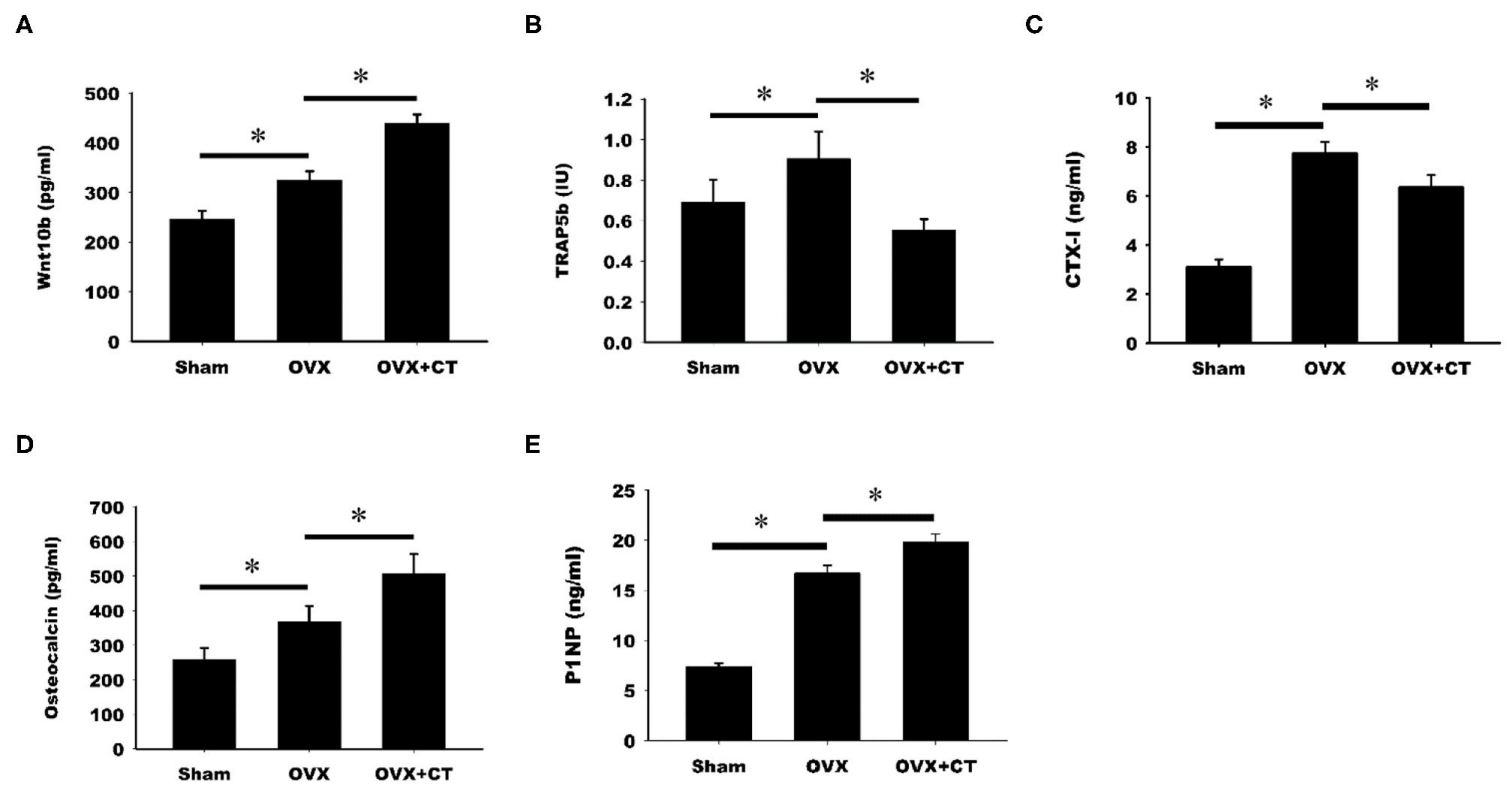
Calcitonin increases Wnt10b and bone formation but decreases bone resorption in ovariectomized osteoporotic rats. Sham-operated rats and ovariectomized (OVX) rats were treated as indicated in Figure 1. Serum samples obtained from rats were analyzed by ELISA. **(A)** Quantitative analysis of Wnt10b **(B)** Quantitative analysis of TRAP5b **(C)** Quantitative analysis of CTX-1 **(D)** Quantitative analysis of osteocalcin **(E)** Quantitative analysis of P1NP. *Indicates a significant difference (*p* < 0.05). *N* = 6 in each group.

The serum levels of Wnt10b and bone formation and resorption markers were analyzed by ELISA. As shown in Figure 2A, a significant increase in Wnt10b was found in OVX rats compared with sham rats. Calcitonin treatment caused a further increase in Wnt10b in OVX rats. Analysis of serum bone resorption markers, TRAP5b and CTX-1, revealed increased bone resorption in OVX rats compared with sham rats (Figures 2B,C). TRAP5b and CTX-1were significantly lower in the calcitonin treatment group compared with the untreated group. Increases in the serum bone formation markers, osteocalcin, and P1NP, were observed in OVX rats compared with sham rats (Figures 2D,E). Calcitonin treatment caused a further increase in osteocalcin and P1NP in OVX rats.

### Calcitonin Treatment Increases the Expression in Metaphysis of Femoral Bone in Ovariectomy-Induced Osteoporotic Rats

**Figure 3:**
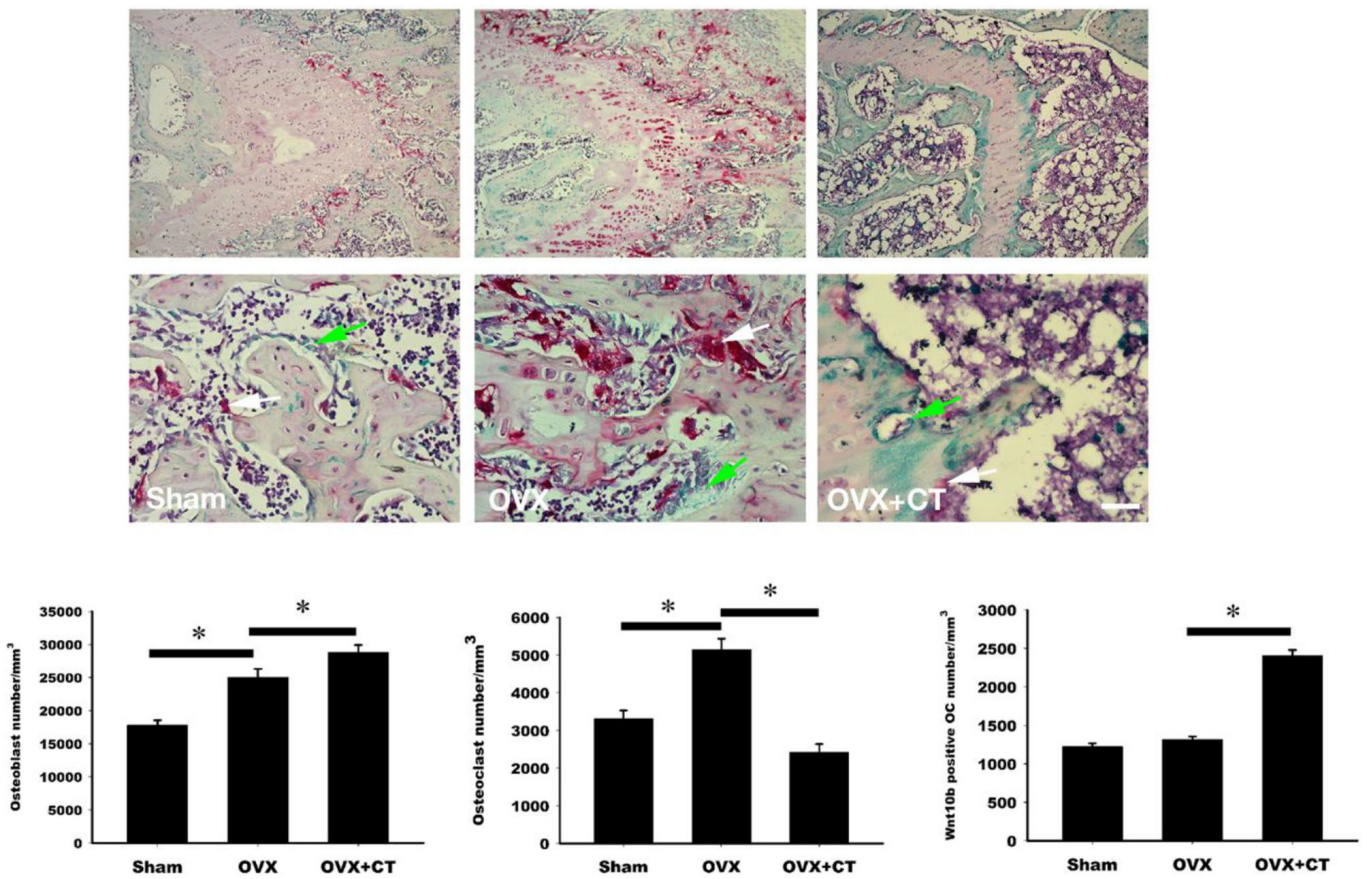
Calcitonin treatment increases the osteoclastic Wnt10b expression in metaphysis of femoral bone in ovariectomy-induced osteoporotic rats. Sham-operated rats and ovariectomized (OVX) rats were treated as indicated in Figure 1. Immunohistochemistry labeling of Wnt10b (green) and TRAP stain (red) were performed near resorption zone of growth plate in femoral bone. Decreased Wnt10b expression was found in OVX rats. CT treatment increased Wnt10b expression. The number of the osteoblast, osteoclast, and osteoclast with Wnt10b were counted. Green arrow showed osteoclasts to secret Wnt10b; White arrow showed osteoclasts that did not secret Wnt10b. Scale bar = 50 μm. *Indicates a significant difference (*p* < 0.05). *N* = 6 in each group.

Immunohistochemistry labeling of Wnt10b was performed in femoral bone (Figure 3). Positive Wnt10b labeling was noted as green near resorption zone of growth plate in femoral bone of sham rats. Decreased Wnt10b and increased TRAP expressions were found in OVX rats as compared with sham rats. In the CT treatment group, increase of Wnt10b and decrease of TRAP expressions were also found near resorption zone of growth plate in femoral bone.

### Calcitonin Increases Wnt10b Expression in Osteoclasts

**Figure 4:**
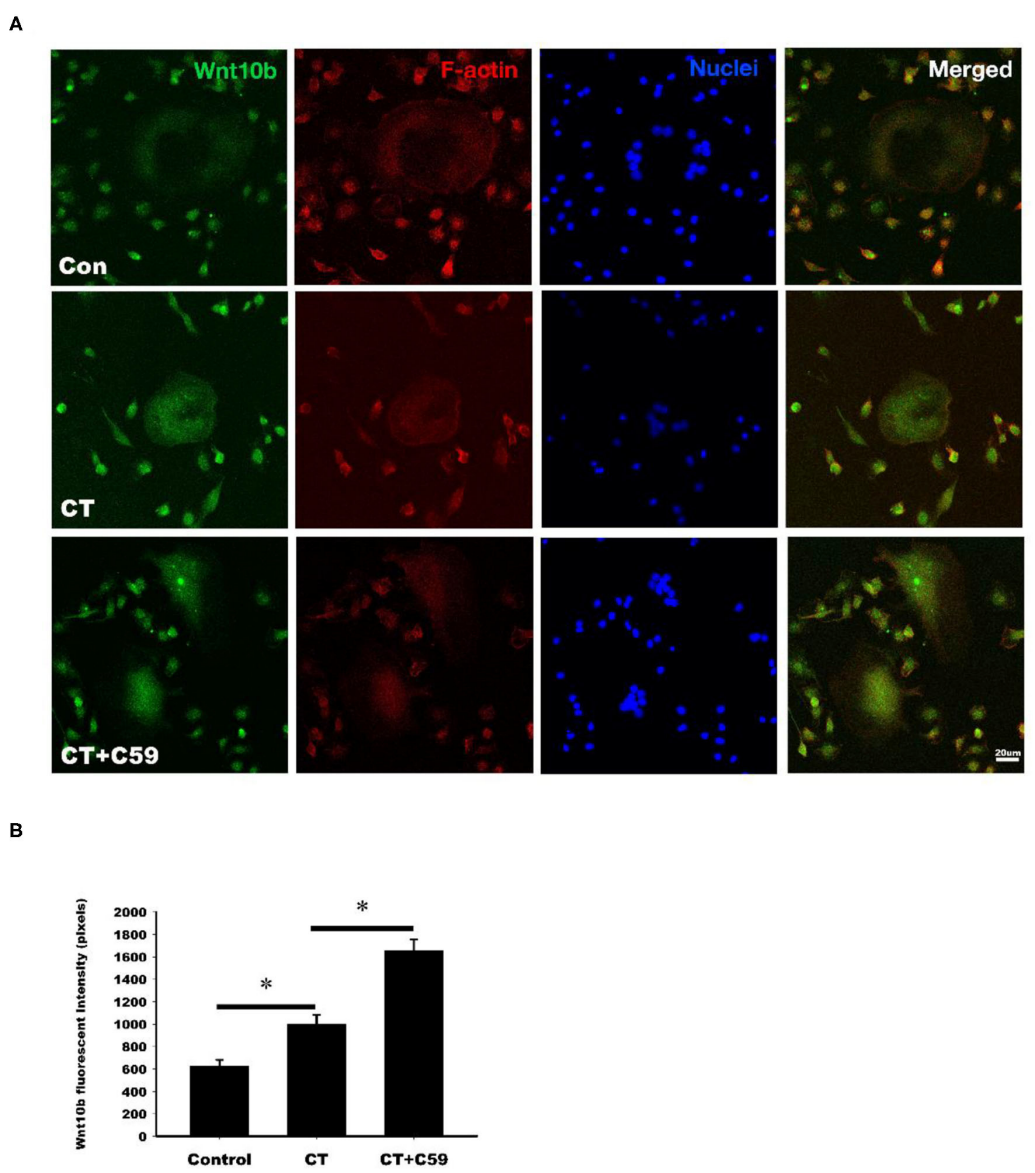
Calcitonin increases intracellular Wnt10b expression in osteoclasts. Bone marrow monocytes were isolated from femoral and tibial bones of 8-week-old SD rats. They were induced into osteoclasts by M-CSF and RANKL stimulation. **(A)** Confocal analysis was performed in osteoclasts treated with 3 nM calcitonin alone or with C59 for 16 h. Osteoclasts were labeled with rhodamine phalloidin (red) to visualize F-actin and Nuclear Red (blue) to visualize nuclei. Scale bar = 20 μm. **(B)** The statistics of Wnt10b fluorescent intensity was showed in each group. *Indicates a significant difference (*p* < 0.05).

Immunofluorescent labeling of Wnt10b was performed in osteoclasts isolated from rat bone marrow. Confocal analysis of immunofluorescently labeled Wnt10b showed that it was greater in osteoclasts treated with calcitonin compared with controls (Figure 4). Pretreatment with C59, a Wnt secretion inhibitor, further increased the calcitonin effect of Wnt10b expression within the osteoclasts and demonstrated that calcitonin increased Wnt10b release from osteoclasts.

**Figure 5:**
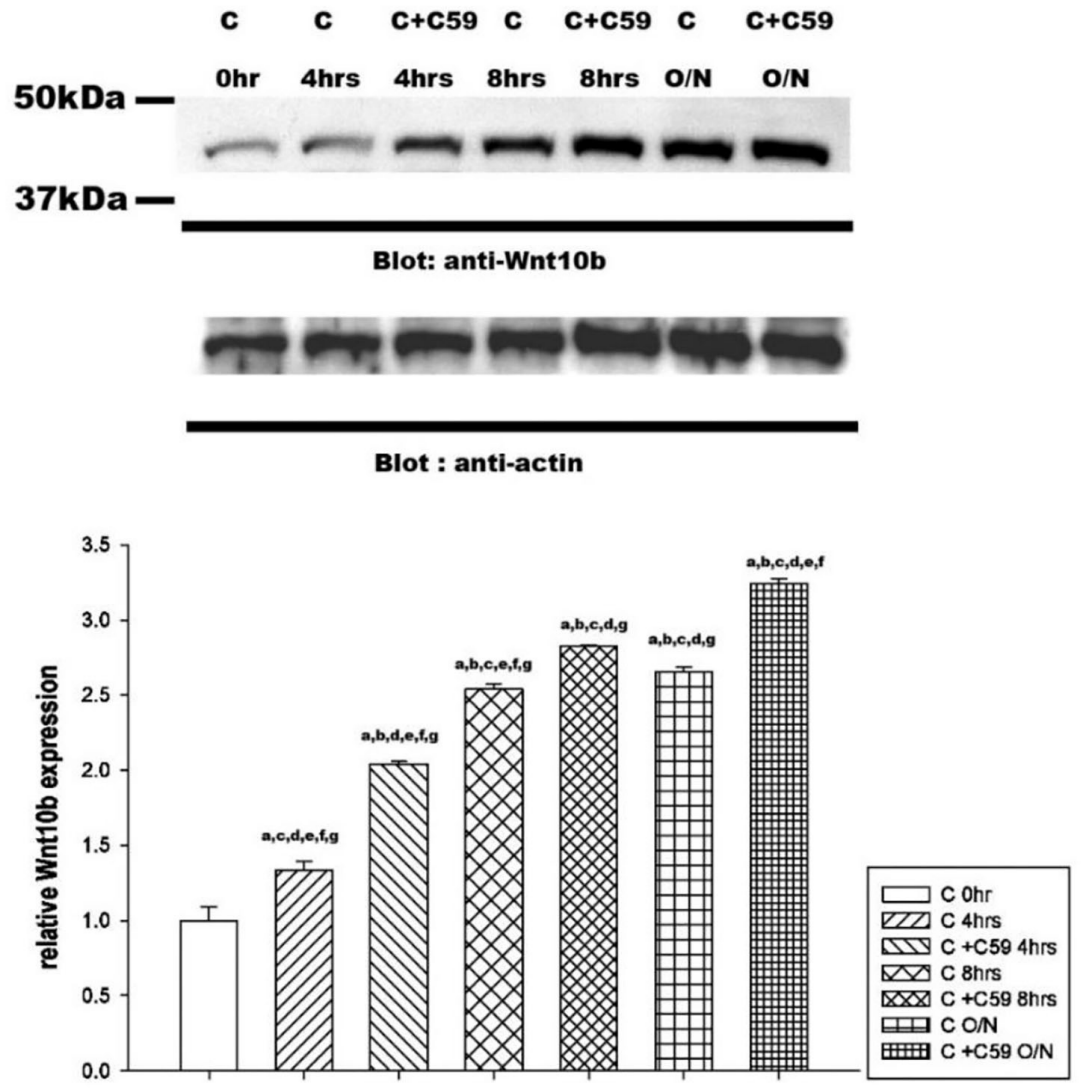
Calcitonin increases Wnt10b levels in osteoclasts. Osteoclasts were prepared and treated as indicated in Figure 4. Western blot analysis was performed on osteoclasts treated with 3 nM calcitonin alone or with C59 for the various times indicated. Protein levels were quantified by densitometry, corrected for sample loading on the basis of actin levels, and expressed as the fold change relative to the control lane. Each blot is representative of at least three replicate experiments.

Western blot analysis revealed a significant time-dependent increase in Wnt10b expression in calcitonin-treated osteoclasts (Figure 5). Pretreatment with C59, a Wnt secretion inhibitor, further enhanced the effect of calcitonin in increasing Wnt10b expression within the osteoclasts.

### Calcitonin-Induced Osteoclastic Wnt10b Secretion Improves Osteoblastic Mineralization

**Figure 6:**
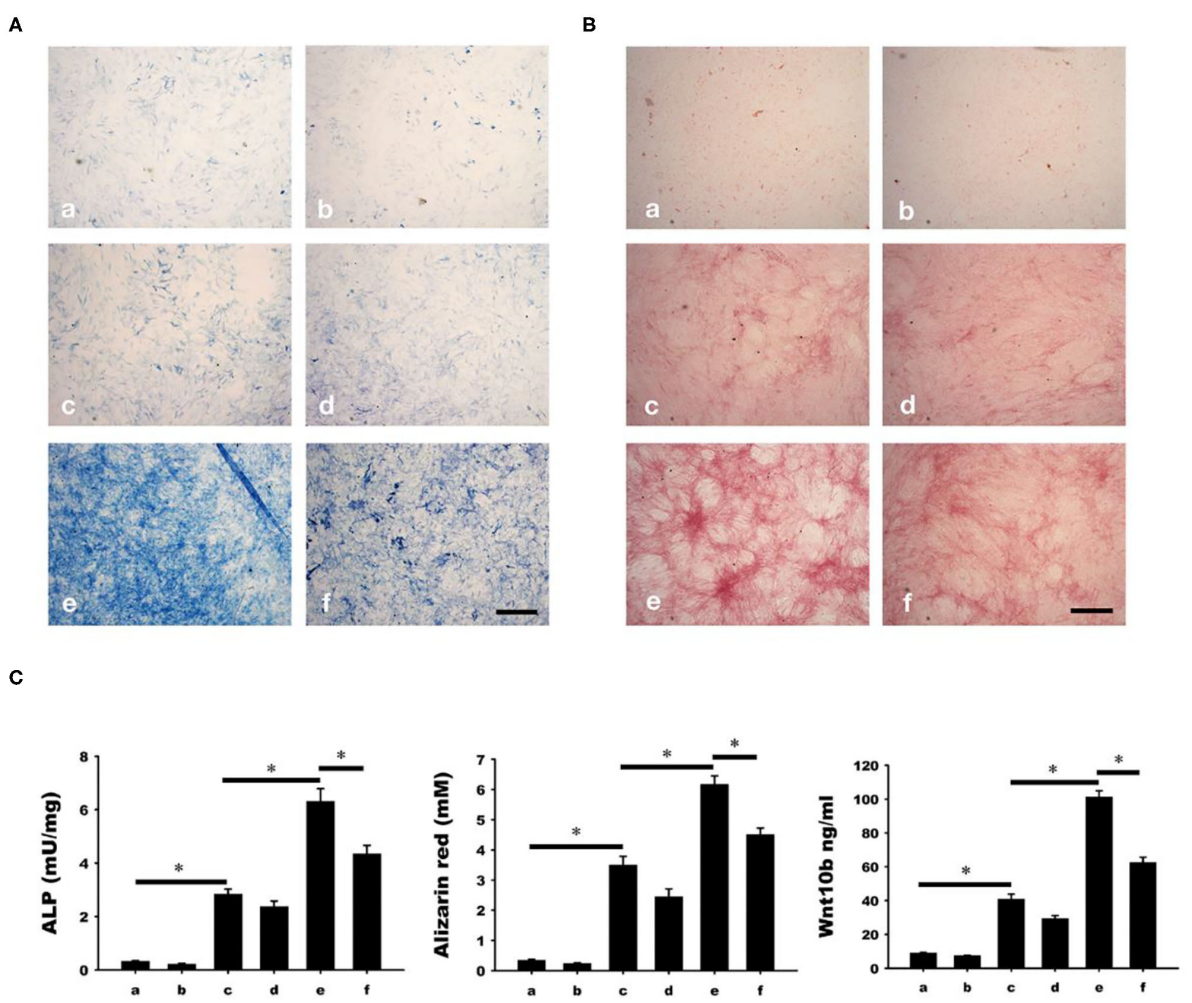
Calcitonin indirectly increases osteoblast mineralization. Osteoblasts were isolated from the calvariae of 1-day-old newborn SD rats and cultured in groups a-f medium as indicated in Materials and Methods. Analysis of osteoblast mineralization was performed by ALP **(A)** and alizarin red staining **(B)**. **(C)** Quantitative results of the experiment shown in **(A,B)**, and Wnt10b concentration in mediums of each group before cultured with osteoblasts. *Indicates a significant difference (*p* < 0.05). Bar = 500 μm.

Osteoblastic mineralization was analyzed by alkaline phosphate (ALP, Figure 6A) and alizarin red (Figure 6B) staining. As a negative control, osteoblasts isolated from the calvariae of 1-day-old rats were cultured in a-MEM with or without C59 (a and b in Figure 6). Significant increase of ALP and alizarin red staining (c and d in Figure 6) were found in osteoblasts cultured in conditioned medium with or without C59. Further increase of ALP and alizarin red staining (e in Figure 6) were found in calcitonin-treated conditioned medium. The calcitonin-induced increase of ALP and alizarin red staining was reduced by C59 cotreatment (f in Figure 6). Similar finding of change of Wnt10b concentration was noted in these groups.

## Discussion

**Figure 7:**
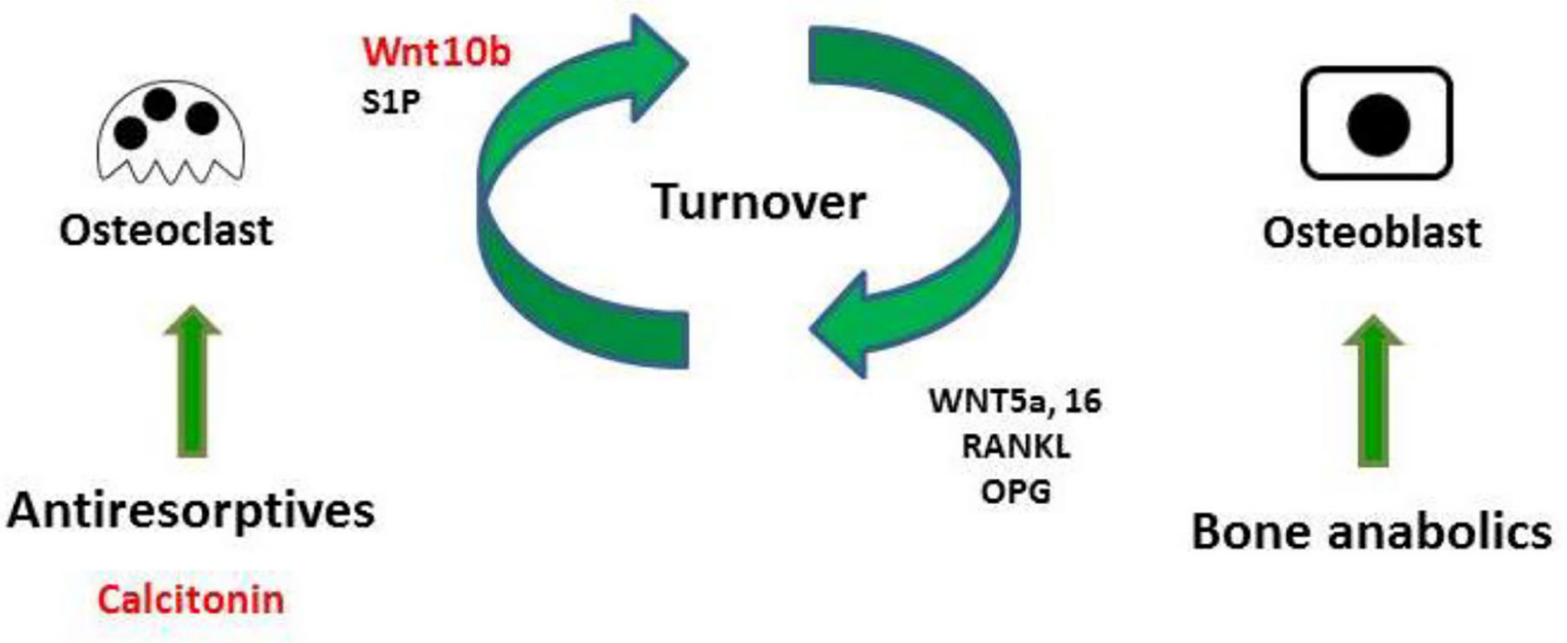
Model of calcitonin-induced bone formation through action on osteoclasts. Bones undergo constant remodeling throughout the lifetime of the organism, and this involves the continuous activity of osteoblasts and osteoclasts. Osteoblastic bone formation is communicated to osteoclastic bone resorption by positive and negative modulators (RANKL/OPG or WNT). Osteoclasts also communicate to osteoblasts by clastokines (Wnt10b and S1P). Bone anabolic such as PTH also increase osteoclastic bone resorption through their control of RANKL/OPG and WNT signaling. Anti-resorptive inhibit osteoclast resorption, which usually causes the inhibition of bone formation. Uncoupling anti-resorptive, such as calcitonin, may modulate osteoblast mineralization through the controlled secretion of Wnt10b and S1P.

The present study used micro-CT analysis to show that calcitonin alleviated bone loss in ovariectomy-induced osteoporotic rats (Figure 1). Though calcitonin is no longer considered an appropriate treatment option for osteoporosis, the effects of calcitonin on the coupling process between osteoclasts and osteoblasts remain uncertain. Thus, it is fundamentally important to uncover the mechanism by which calcitonin affects osteoblastic bone formation through its actions on osteoclasts (Figure 7).

Consistent with previous studies, we found that calcitonin treatment not only decreased the levels of serum bone resorption markers (i.e., TRAP5b and CTX-1) in OVX rats (Figure 2) (14, 15) but also led to increased levels of bone formation markers (i.e., osteocalcin and P1NP) in OVX rats (11, 15). Because Wnt10b has recently been identified as a clastokine able to increase osteoblast activity, the finding that increased Wnt10b serum levels are correlated with osteocalcin and Wnt10b expression in bone marrow in calcitonin-treated OVX rats (Figure 3) implies that Wnt10b may be involved in the effects of osteoclasts coupling to osteoblasts. Confocal microscopy of immunofluorescently labeled Wnt10b (Figure 4) and Western blot analysis of Wnt10b expression (Figure 5) in osteoclasts provided additional evidence to support this hypothesis. Moreover, osteoblastic mineralization was enhanced in conditioned medium derived from calcitonin-treated osteoclasts (Figure 6). Taken together, these results demonstrate that calcitonin induces bone formation by increasing the expression of Wnt10b in osteoclasts in ovariectomy-induced osteoporotic rats (Figure 7).

It is well-documented that Wnt signaling plays a crucial role in many biological processes (e.g., cellular proliferation, tissue regeneration, and other systemic effects) (16). This is because the Wnt family is characterized by at least 19 different glycoproteins, each of which is responsible for triggering multiple signaling cascades. Accordingly, Wnt proteins are likely to be involved in various aspects of bone biology, including osteoblastic and osteoclastic functions and endochondral bone formation. A study indicated that Wnt signaling plays a vital role in osteoblast differentiation from both mesenchymal precursors and osteochondo progenitors as well as the proliferation and survival of osteoblasts (17). Wnt ligands have been widely studied by means of various osteoblastic models and, later, animal models. Currently, existing murine models suggest that Wnt3a, Wnt5a, and Wnt10b are critical for osteoblast regulation, whereas Wnt14 is important for endochondral bone formation (18–20). Further investigations in this research area are necessary to comprehend the scope of Wnt effects on bone metabolism and the effectiveness of Wnt-based therapeutics on bone structure and functions.

In contrast to the numerous findings regarding Wnt signaling in osteoblast lineage cells, little is known about the influence of Wnt proteins on osteoclasts in the context of cell autonomy. It has been proposed that matrix-bound TGF-β1 could function as an effective coupling agent for actively recruiting osteoblast-lineage cells to bone-resorption positions following its osteoclast-mediated release. A study indicated that TGF-β1 improves the coupling to osteoblasts by inducing Wnt10b expression in osteoclasts (21). Moreover, researchers have suggested that cinacalcet, probably via increased bone mineralization related to osteoclastic Wnt10b secretion, might improve bone quantity, and quality in chronic kidney disease (CKD) mice (22). Moreover, in a study involving calcitriol treatment of secondary hyperparathyroidism in CKD patients, it was demonstrated that the increased secretion of osteoclast-derived Wnt10b was critical for the improvement of bone anabolism through its inhibition of osteoclastogenesis and promotion osteoblastogenesis (23). Hence, Wnt10b is a clastokine and a potential novel therapeutic target of postmenopausal osteoporosis and CKD-related bone disorder. On the other hand, runx2 directly induces Wnt10b expression in osteoblasts (24). PTH is a bone anabolic agent and effective treatment for postmenopausal osteoporosis, maybe by its effect of increase Wnt10b production in osteoblasts (25). Therefore, the role of osteoblast-derived Wnt10b could not be underestimated.

The elemental mechanisms of calcitonin receptor (CTR) bone activity were discovered by Keller et al. (8) through experiments on a new strain of viable global Calcr KO mice, which were generated by applying a specific technique to knockout the expression of CTR. Briefly, the study asserted that losing CTR in osteoclasts would increase the levels of sphingolipid transporter 2 (spinster 2, SPNS2), an exporter protein required for the secretion of sphingosine-1-phosphate (S1P), which can effectively induce bone formation. Thus, on the basis of the above mechanisms, calcitonin binding to CTR on osteoclasts will inhibit SPNS2 expression, causing the decreased secretion of S1P and subsequent inhibition of osteoblast activity. Because calcitonin-stimulated osteoclasts could inhibit or stimulate osteoblast functions through the modulation of different cytokines, the roles that these cytokines may play in various stages of bone resorption remain unknown. Consequently, further studies are needed to dissect the underlying mechanisms and corresponding physiological relevance during bone remodeling.

However, C59 is a Wnt inhibitor, not specific for Wnt10b. This is a limitation in our experiment. In addition, osteoblasts may also secrete Wnt10b which is interesting issue worth of more study. The current clinical application of calcitonin is gradually decreasing because the other long-acting drugs for inhibition of bone resorption are effective and convenient. Many studies still have a great interest in calcitonin and the effect of the bone metabolism, physiological roles, and the activities. This is an intricate and fantastic peptide.

## Materials and Methods

### Ovariectomy-Induced Osteoporosis RAT Model

All rat experiments were reviewed and approved by the Institutional Animal Care and Use Committee (IACUC) of the Laboratory Animal Center of the National Defense Medical Center; the identification number is IACUC-14-104. Briefly, 18 4-month-old female Sprague–Dawley (SD) rats were purchased from a specific pathogen-free laboratory animal company (BioLASCO, Taipei, Taiwan) and separated randomly into three groups. All rats were acclimatized under accepted laboratory conditions (temperature was 22 ± 2°C, and humidity was 50 ± 10%). Food and water were provided ad libitum. Rats were anesthetized by the administration of isoflurane (Forane® AbbVie Inc., Queenborough, UK), and bilateral ovariectomy (OVX) was performed to build an osteoporosis model in OVX and OVX calcitonin-treated groups. The sham control group comprised rats whose ovaries were exposed but not removed. Twenty-eight days after surgery, the following three groups (6 rats per group) were set up: (a) sham control group: rats underwent a sham operation and were subcutaneously injected with the same volume of normal saline; (b) OVX group: rats underwent the OVX operation and were subcutaneously injected with the same volume of normal saline; (c) OVX-calcitonin group: rats underwent the OVX operation and were subcutaneously injected with calcitonin (5 IU/kg/day, Miacalcic, NovartisPharma). All groups were treated five times per week for 4 weeks. At the endpoint of the experiment, blood and femurs were obtained from the rats, which were first fasted overnight and euthanized, coded, and prepared for blinded distribution. Collected sera were frozen at −80°C, and femurs were kept in alcohol at 4°C.

### Micro-Computed Tomography

The micro architecture of the 18 femoral trabecular bones was investigated using micro-computed tomography (Skyscan 2211 Nanotomograph Micro-CT; Skyscan, Aartselaar, Belgium) at a resolution of 8.5 μm. The scan was performed with 180° scanning at a voltage of 80 kVp and a current of 500 μA (7.9 W output). Image reconstruction was performed using GPU-based reconstruction software, GPU-Nrecon. Ring artifacts and beam-hardening corrections were also performed using this software. Reconstructed cross-sections were reorientated, and the region of interest (ROI) was further selected. We performed the analysis of the secondary trabecular bone area using 2 mm (236 slices) images. The volume of interest was 1.5–3.5 mm below the growth plate. Thresholding, region of interest selection, and bone morphometric analysis were performed using CTAn software. The volume of interest was selected as 1.5–3.5 mm below the growth plate. In addition, the region of interest (ROI) of the trabecular bone area was selected and then analyzed by using CTAn software.

### Biochemical Analyses

The concentrations in sera of Wnt10b and the bone resorption marker TRAP5b (tartrate-resistant acid phosphatase from 5b) were measured by ELISA (Wnt 10b (MBS2533600, MyBioSource, San Diego, CA, USA), TRAP5b (SB-TR102, Immunodiagnosticsystems, East Boldon, UK), and resorption marker CTX-1 (C-telopeptide of type I collagen) and formation markers osteocalcin and P1NP (type 1 procollagen amino-terminal—propeptide) were measured EIA (AC-06F1, AC-12F1, and AC-33F1 Immunodiagnosticsystems, UK).

### Immunohistochemistry and TRAP Double Stain of the Femur

Femurs were taken from experimental rats, then washed several times in PBS and fixed overnight in formalin substitute solution (FX1075, Cancer Diagnostics, Durham, NC, USA). The femurs were decalcified in Decalcifying Solution (REF. No.3840, Thermo Scientific, Waltham, MA, USA) for 24 h, then transferred to 0.5 M EDTA, pH 8, for several days until a biopsy needle could be inserted in to the femur. After decalcification, tissues were dehydrated, embedded in paraffin, and cut into 10 μm thickness per slices. After deparaffinization, rehydration, and antigen retrieval and blocking, anti-Wnt10b Ab (NBP2-49165, Novus Biologicals, Centennial, CO, USA) was applied to the slides followed by incubation overnight. The prepared slides were then developed with the substrate chromogen of HRP-green (TA01HG-15, BioTnA, Kaohsiung, Taiwan). After chromogen properly developing the TRAP staining (AK04F-COS, COSMO BIO, Tokyo, Japan) was applied. Briefly, the chromogen was mixed with buffer immediately. Finally, hematoxylin was applied to show the nuclei.

### Osteoclast Differentiation From Bone Marrow-Derived Monocytes

After 8-week-old Sprague-Dawley (SD) rats were sacrificed, bone marrow from the tibiae and femurs were collected in 0.05% citric acid normal saline. Harvested cells (contained monocytes) from bone marrow were extracted by centrifugation in an equal volume of Ficoll-Paque PLUS (17144002, GE Healthcare, Chicago, IL, USA). A total of 10^6^ harvested cells were cultured in a 10 cm dish with osteoclast differentiation medium (α-MEM medium containing 10% fetal bovine serum (FBS, SH30088.03, GE Healthcare, USA), 50 ng/mL macrophage colony-stimulating factor (M-CSF, 400-28, PEPROTECH, Rocky Hill, NJ, USA), 50 ng/mL RANKL (315-11, PEPROTECH, USA), and 1% Antibiotic-Antimycotic Solution (30-004-CI, CORNING, Corning, NY, USA). The medium was changed every 3 days.

### Analysis of the Distribution of Wnt10b in Osteoclasts by Confocal Microscopy

Osteoclasts induced from bone marrow-derived monocytes were cultured on 22 × 22 mm glass coverslips in the medium described above. After the osteoclasts formed, they were separated into three groups: the control group, the group treated with 3 nM calcitonin (05-23-2401,Sigma-Aldrich, St. Louis, MO, USA), and the group treated with 3 nM calcitonin combined with a Wnt inhibitor, C59 (sc-475634, SANTA CRUZ BIOTECHNOLOGY); all the groups were cultured with α-MEM medium containing 10% FBS and 1% Antibiotic-Antimycotic Solution for 24 h. C59 was added in the medium in control group. After the osteoclasts were washed with PBS, fixed with formalin substitute, and permeabilized with 0.1% Triton X-100 in PBS for 10 min, the cells were blocked in PBS containing 10% normal rabbit serum (AR1010, BOSTER, Pleasanton, CA, USA) for 1 h at room temperature. Then, anti-Wnt10b Ab was applied over night at 4°C. On the second day, after washing in 0.05% Triton X-100 PBS three times, anti-rabbit Ab conjugated FITC was applied at room temperature for 2 h. F-actin was labeled with F-Actin Labeling Kit (22663, AAT Bioquest, Sunnyvale, CA, USA), and nuclei were stained with Nuclear Red DCS1 (157199-63-8, AAT Bioquest, USA). The distribution and intensity of Wnt10b was analyzed using a confocal microscope (LSM 510, Zeiss, Oberkochen, Germany).

### Quantitation of Wnt10b in Osteoclasts by Western Blot

The total protein was extracted from lysed osteoclasts treated with or without calcitonin and C59 in a lysis buffer (100 mM Tris-HCl, pH7.4, 1% NP-40) containing protease inhibitor cocktail (F1PICo25, Bio Future, New York, NY, USA). The proteins were separated by 10% SDS-PAGE and transferred to a PVDF membrane. After blocking, the membrane was incubated with primary Ab overnight at 4°C. The next day, after washing, the secondary Ab was applied at room temperature for 2 h. Finally, the membrane was treated with electrogenerated chemiluminescence reagent (RPN2235, GE Healthcare, USA) and detected using UVP ChemStudio PLUS (849-97-0851-03, Analytik Jena, Jena, Germany).

### Rat Calvariae Osteoblast Separation

The calvariae of newborn rats were cut into chips and digested in a digestive solution of α-MEM medium containing 1% type 2 collagenase (LS004174, Worthington, OH, USA) and 0.05% trypsin for 30 min. After removing the solution, fresh digestive solution was added to digest the calvariae chips; this process was repeated three times. Finally, after the fourth time, centrifuged osteoblasts, and calvariae chips were cultured in α-MEM medium containing 10% FBS and 1% Antibiotic-Antimycotic Solution.

### Conditioned Medium Culture of Osteoblasts

Osteoblasts separated from rat calvariae were cultured in group a: osteoblast culture medium alone as mentioned in 4.8; group b: osteoblast culture medium with C59; group c: half of osteoclast culture medium as mentioned in 4.5 (conditioned medium) and half of osteoblast culture medium with 50 mg/mL ascorbic acid (A1968, Sigma Aldrich, USA) and 2 mM b-glycerophosphate (G9422, Sigma Aldrich, USA) (bone formation medium); group d: half of conditioned medium with C59 and half of bone formation medium; group e: half of 3 nM calcitonin-treated conditioned medium and half of bone formation medium; group f: half of 3 nM calcitonin-treated conditioned medium with C59 and half of bone formation medium. For the ALP stain and ALP activity assay, each group was cultured for 7 days. For the alizarin red stain, each group was cultured for 18 days. Before osteoblast were cultured, the concentration of Wnt10b in each group of medium were detected by ELISA (MBS2533600, MyBioSource, San Diego, USA).

### ALP Staining and ALP Activity Assay of Osteoblasts

Osteoblasts were stained with an ALP staining kit (AK20-COS, COSMO BIO, Tokyo, Japan). Briefly, for staining, osteoblasts were fixed by formalin substitute solution. The buffer and the substrate were mixed, and the solution was then applied to coverslips containing osteoblasts at room temperature for 20 min, followed by washing with deionized water to stop the reaction. The images were captured by microscopy (Axio Imager A2, Zeiss, Germany). Quantified ALP activity in osteoblasts was measured using an Alkaline Phosphatase Assay Kit (ab83369, Abcam, Cambridge, UK). Briefly, cells were lysed in lysis buffer and combined with p-nitrophenyl phosphate (pNPP) as a phosphatase substrate, then incubated at room temperature for 1 h. Finally, the reaction was stopped by the addition of 0.1 M NaOH solution, and the ALP activity was estimated by the optical absorbance measured at 405 nm.

### Alizarin Red S Staining and Quantification

Osteoblasts were cultured for 18 days and stained with Alizarin Red S (0223, ScienCell, Carlsbad, CA, USA) at room temperature for 20 min, and images were captured after washing. To quantify calcium mineralization in cells, the stained material in cells was dissolved in 10% cetylpyridinium chloride (C0732, Sigma-Aldrich, USA) at room temperature for 1 h, and the quantification was estimated by the optical absorbance measured at 405 nm.

### Statistical Analysis

Each series of experiments was repeated at least three times. The results obtained from a typical experiment were expressed as the means ± S.D. Significant differences were determined using factorial analysis of variance. Group comparisons were made by one-way ANOVA followed by the Dunnett's test using SPSS software, version 15.0 (Armonk, NY, USA).

## Conclusions

Using *in vivo* OVX rats and *in vitro* osteoclast and osteoblast cultures, we show that calcitonin induces bone formation by increasing the expression of Wnt10b in osteoclasts in ovariectomy-induced osteoporotic rats. The present study provides further information about calcitonin at the molecular level of bone remodeling, and will thus help in future potential therapeutic studies on postmenopausal osteoporosis.

## Data Availability Statement

The raw data supporting the conclusions of this article will be made available by the authors, without undue reservation.

## Ethics Statement

The animal study was reviewed and approved by the Institutional Animal Care and Use Committee (IACUC) of the Laboratory Animal Center of the National Defense Medical Center; the identification number is IACUC-14-104.

## Author Contributions

J-FS, C-YH, T-HChe, and P-JT: study conception and design. T-HChe, C-YH, J-FS, Y-NT, and T-HChu: methodology and investigation. C-YH: formal analysis. J-FS and T-HChe: resources. T-HChu, J-FS, and P-JT: writing—original draft preparation. C-YH, P-JT, T-HChe, and J-FS: writing—review and editing. C-YH, J-FS, and T-HChe: supervision. C-YH, P-JT, T-HChe, and J-FS: project administration. All authors have read and agreed to the published version of the manuscript.

## Conflict of Interest

The authors declare that the research was conducted in the absence of any commercial or financial relationships that could be construed as a potential conflict of interest.

## Abbreviations

ALP: alkaline phosphate
CKD: chronic kidney disease
CTX-1: type 1 carboxyterminal collagen fragments
Calca: calcitonin
Calcr: calcitonin receptor
CT: computer tomography
M-CSF: macrophage colony-stimulating factor
OVX: ovariectomy
P1NP: amino-terminal propeptide of type 1 procollagen
RANKL: receptor activator of nuclear factor kappa B ligand
S1P: sphingosine-1-phosphate
TGF-β1: transforming growth factor-beta 1
TRAP5b: tartrate-resistant acid phosphatase 5b.
